# Exosomes as Central Mediators of Host-Pathogen Interactions in Periodontitis: A Systematic Literature Review

**DOI:** 10.7759/cureus.95609

**Published:** 2025-10-28

**Authors:** Goda Marija Dabkeviciute, Albertas Kriauciunas

**Affiliations:** 1 Faculty of Odontology, Lithuanian University of Health Sciences, Kaunas, LTU; 2 Clinic of Dental and Maxillofacial Orthopedics, Lithuanian University of Health Sciences/Hospital of Lithuanian University of Health Sciences, Kaunas, LTU

**Keywords:** cell behaviour, exosomes, immune cells, inflammatory factors, mrna, periodontitis

## Abstract

Periodontitis is a chronic inflammatory disease that compromises the periodontium and is among the most prevalent oral conditions worldwide. Recent studies highlight exosomes - small extracellular vesicles carrying nucleic acids, lipids, metabolites, and proteins - as key mediators of intercellular communication contributing to disease progression. The objective of this review was to systematically analyze the existing scientific literature regarding the role of exosomes in the pathogenesis of periodontitis. This systematic review was conducted in accordance with the Preferred Reporting Items for Systematic Reviews and Meta-Analyses (PRISMA) protocol. The study question was formulated using the Population, Intervention, Comparison, and Outcome (PICO) framework: “Do exosomes contribute to the pathogenesis of periodontitis?”. A literature search was performed in PubMed and ScienceDirect databases for articles published between 2015 and 2025. Inclusion criteria were restricted to original research, written in English, involving human participants or human-derived samples that explicitly focused on exosomes in the context of periodontitis. Out of 1,000 identified records, 106 full-text articles were screened, and six met the inclusion criteria. The included studies investigated salivary, gingival, or periodontal ligament stem cell-derived exosomes and reported their roles in pyroptosis, macrophage polarization, angiogenesis, and immune modulation. Key findings demonstrated that the downregulation of exosomal miR-223-3p enhanced NLRP3-mediated pyroptosis, while exosomal miR-143-3p promoted M1 macrophage polarization via the PI3K/AKT/NF-κB signaling pathway. In addition, exosomal VEGFA regulated by miR-17-5p promoted angiogenesis, and salivary exosomes exhibited immune-related protein cargo, decreased tetraspanins (CD9, CD81), and elevated PD-L1 mRNA in advanced disease. Collectively, this review underscores the ability of exosomes to transport diverse molecular cargo and influence recipient cell behavior, highlighting their role as mediators of intercellular communication in periodontal inflammation.

## Introduction and background

Periodontitis is a chronic inflammatory disease affecting more than 60% of the adult population [1]. By targeting the periodontium, a complex structure formed by gingiva, cementum, periodontal ligament, and alveolar bone, periodontitis compromises the stability of the dentition [2,3]. As the disease progresses, periodontal structures undergo pathological changes, which manifest clinically as gingival bleeding, inflammation, gum recession, and, in advanced stages, increased tooth mobility, which can eventually lead to tooth loss [4]. 

Periodontitis is considered a dysbiosis-driven disease rather than a classical infection caused by one or a few specific bacterial species [5]. Keystone pathogens disrupt host-microbe homeostasis by subverting immune responses, thereby triggering the release of pro-inflammatory cytokines, including interleukins such as IL-1, IL-6, and IL-23, which activate neutrophils, T cells, and fibroblasts [6]. As a consequence, the immune response upregulates receptor activator of NF-κB (RANK) ligand expression, a signaling molecule that promotes osteoclast differentiation and bone resorption, and matrix metalloproteinase (MMP) production, a group of zinc-dependent enzymes responsible for extracellular matrix degradation, ultimately resulting in the destruction of periodontal structures [7-9]. Among them, MMP-8, a collagenase that primarily degrades type I and III collagen, has been identified as a key biomarker of periodontal tissue breakdown, with elevated salivary levels reflecting disease activity and severity [9]. 

While the contribution of immune mediators, such as cytokines, RANKL, and MMPs, is well-established in the pathogenesis of periodontitis, recent attention has shifted toward alternative mechanisms of intercellular communication [10]. In this context, exosomes have emerged as pivotal mediators due to their ability to transport a variety of biologically active molecules, including nucleic acids, lipids, metabolites, and proteins, that reflect the composition of their cell of origin [11,12]. Exosomes influence the behavior of recipient cells and help coordinate local and systemic responses. They are secreted by a wide variety of cells, including immune cells such as macrophages and dendritic cells, mesenchymal stem cells, fibroblasts, epithelial cells, mast cells, lymphocytes, and various tumor-derived cells [13-15]. These nanovesicles, ranging from 30-150 nm in size, are released into the extracellular space when multivesicular bodies fuse with the plasma membrane [16]. Unlike microvesicles or apoptotic bodies, exosomes originate from the endosomal pathway and selectively package molecular cargo reflecting their parent cell, enabling precise and targeted intercellular communication [16,17]. Once released, exosomes can traverse intercellular junctions and migrate beyond their compartment of origin, enabling direct interaction with neighboring cells through membrane fusion or uptake. They have been identified in various biological fluids, including plasma, serum, urine, cerebrospinal fluid, breast milk, saliva, and cell culture supernatants, underscoring their systemic circulation and functional versatility [17,18]. 

Functionally, exosomes regulate a wide range of physiological and pathological processes, including immune responses, inflammation, tumor progression, and infection [19]. In the context of periodontitis, they are thought to contribute to disease development by modulating local immune and inflammatory pathways [17]. Some studies describe exosomes' role in promoting anti‐inflammatory outcomes by enhancing M2 macrophage polarization and dampening cytokine signals through pathways such as ER stress and the unfolded protein response, IL‑6/JAK2/STAT3, and TGFβ1 [20-22]. Various studies report pro‐inflammatory effects that elevate mediators, including TNF‑α, IL‑1β, IL‑6, and IL‑17A via signaling cascades like SASP and PI3K/AKT/NF‑κB [23-25]. Other investigations describe regulation of immune balance through a T helper cell 17 (Th17)/regulatory T cell (Treg) axis, where pro-inflammatory Th17 and anti-inflammatory Treg cells maintain a balance crucial for preventing excessive immune activation, which is mediated by a miR-155-5p/SIRT1 pathway [26,27]. 

As evidence continues to grow regarding exosomes' immunoregulatory functions, it is essential to further elucidate their involvement in the underlying mechanisms of periodontal pathology. Therefore, the aim of this research was to analyze the current scientific literature regarding the role of exosomes in the pathogenesis of periodontitis.

## Review

Materials and methods

Aim of the Systematic Review

This literature review aimed to examine current scientific findings on the role of exosomes in mediating host-pathogen interactions and their contribution to the pathogenesis of periodontitis.

Inclusion and Exclusion Criteria

We predefined a thorough set of inclusion and exclusion criteria to guarantee methodological rigor (Tables 1-2).

**Table 1:** Inclusion criteria.

Inclusion Criteria:
Research involving human participants diagnosed with periodontitis or using human-derived samples from individuals with periodontitis.
Studies investigating exosomes and their impact on periodontitis pathogenesis.
Articles focusing on exosomes or extracellular vesicles are clearly distinguished from other vesicles in relation to periodontitis.
Articles that are written in English and were published within the last ten years

**Table 2:** Exclusion criteria.

Exclusion Criteria:
Non-original articles such as literature reviews, systematic reviews, meta-analyses, case reports, conference abstracts, editorials, letters, or comments.
Studies exclusively involving animal models.
Studies that exclusively address therapeutic, regenerative, or drug-delivery applications of exosomes, without emphasis on their modulatory role.
Studies that do not explicitly focus on exosomes or clearly distinguish them from other extracellular vesicles.
Research focusing on diseases other than periodontitis.
Articles that are written in languages other than English and are older than ten years.

Information Sources and Search Strategy

A systematic literature search was conducted according to the Preferred Reporting Items for Systematic Reviews and Meta-Analyses (PRISMA) [28]. The initial search was performed in May 2025 and included studies published within the last five years (2020-2025) in the electronic databases PubMed and ScienceDirect. The search strategy included the keywords “exosomes” and “periodontitis,” combined with the Boolean operator AND “periodontitis AND exosomes”. However, as the number of eligible studies was insufficient to meet the aims of this review, the search period was subsequently expanded to include studies published from 2015 to 2025. All other inclusion and exclusion criteria remained unchanged. The literature search was limited to publications from 2015 to 2025 to capture studies conducted under contemporary methodological and conceptual frameworks. This period follows the publication of the International Society for Extracellular Vesicles (ISEV) position statements (2014, 2018), which standardized definitions, isolation techniques, and characterization criteria in extracellular vesicle research, thereby ensuring that the included studies reflect current scientific rigor and reproducibility standards [29].

The studies selected for this review underwent a systematic screening process: articles were first identified based on title relevance, duplicates were removed, and the remaining abstracts were screened for alignment with the inclusion criteria. Eligible full-text articles were subsequently assessed in detail to confirm compliance with the defined inclusion and exclusion criteria. Data was then collected. Full-text articles were accessible for all studies included in the screening and eligibility phases, and none were excluded due to limited access.

To define the focus of this review, the study question was formulated using the PICO (Population, Intervention, Comparison, Outcome) framework [30]. The PICO question guiding this review was: “Do exosomes contribute to the pathogenesis of periodontitis?”

Study Selection

**Figure 1:**
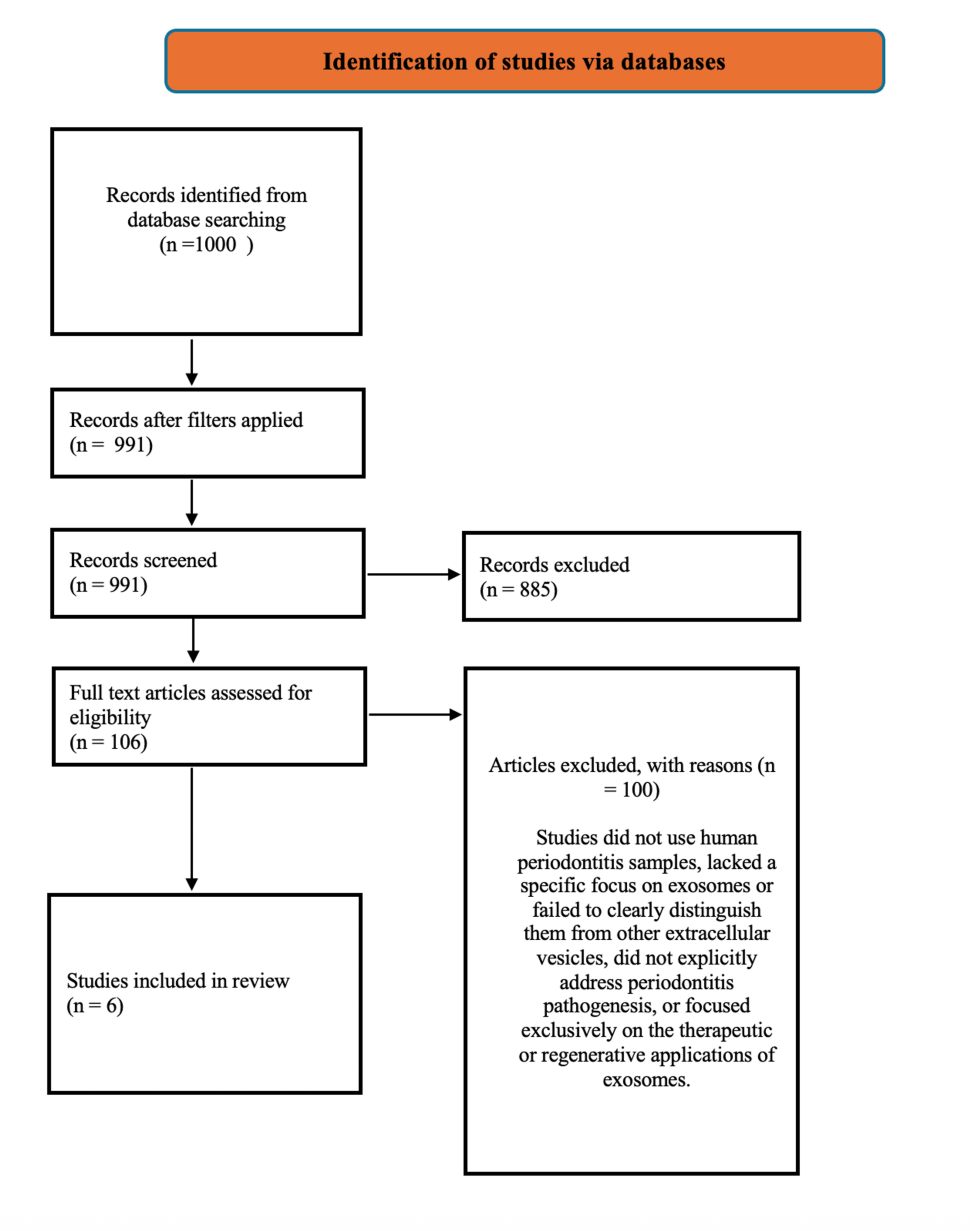
PRISMA flow diagram of the study selection process.

This systematic review was conducted following the PRISMA 2020 guidelines [25]. A total of 1,000 records were initially identified through database searching. After applying filters, 991 articles underwent title and abstract screening, of which 885 were excluded due to irrelevance. Subsequently, 106 full-text articles were retrieved and assessed for eligibility. Following full-text review, 100 studies were excluded for reasons including lack of human samples with periodontitis, lack of specific focus on exosomes, or failure to differentiate exosomes from other extracellular vesicles, or those exclusively investigating therapeutic or regenerative roles of exosomes. Ultimately, six studies were included in the review (Figure 1). Although some included studies reported both in vitro and in vivo (animal) findings, only data derived from human cell-based experiments were extracted and analyzed in this review. Animal experimental data were excluded from analysis. 

Characteristics of the Studies

All studies involved clinically diagnosed periodontitis patients and healthy controls [25,31-35]. However, some studies focused on specific patient subgroups: Yu et al. included patients with mild, moderate or severe periodontitis based on the 2007 American Academy of Periodontology classification [35], Xia et al. analyzed samples from individuals with stage III/IV periodontitis in accordance with the criteria described in their study [31], Huang et al. focused on young adults with severe periodontitis classified according to Armitage 1999 [32]. Tobón-Arroyave et al. classified periodontitis patients based on the 2017 World Workshop criteria [34].

Four studies analyzed salivary exosomes [31,32,34,35], two of these used only saliva [32,34], while the other two included both saliva and gingival tissue [31,35]. Two studies used periodontal ligament stem cells (PDLSC)-derived exosomes [25,33]. 

Two studies focused on exosomal protein [32,34]. Two studies investigated microRNA-mediated immune modulation: both focused on miRNA carried by exosomes, but Wang et al. examined M1 macrophage polarization, while Xia et al. focused on pyroptosis activation [25,31]. Only one study specifically evaluated the levels of mRNA (PD-L1) in salivary exosomes from periodontitis patients [35]. One study investigated VEGFA regulation and angiogenesis [33].

Exosome isolation and characterization methods were broadly consistent across studies. Two studies isolated exosomes using sequential centrifugation and ultracentrifugation [25,33]. Four studies used commercial exosome isolation kits in combination with centrifugation [31,32,34,35]. 

Exosome morphology was characterized using transmission electron microscopy (TEM) in all six studies [25,31-35], while nanoparticle tracking analysis (NTA) was also used in two studies to determine vesicle size [31,35]. Immunoblotting for exosomal markers was performed in five studies [26,31-33,35], whereas one study quantified exosomal tetraspanins by ELISA [34]. Proteomic profiling of exosomal cargo using LC-MS/MS was performed by one study [32]. GO and KEGG pathway enrichment analyses were performed in one study to categorize exosomal proteins and explore their biological functions [32], while KEGG analysis was used in another study to investigate predicted miRNA targets [25]. Quantitative real-time PCR (qRT-PCR) was used for miRNA quantification and mRNA analysis in three studies [25,31,33]. One study conducted qRT-PCR solely for mRNA quantification [35]. Three included studies utilized human THP-1 cell lines; two of these studies used THP-1-derived macrophages to investigate inflammatory and pyroptotic pathways [25,31], while one study used THP-1 cells as a positive control for immunoblotting [35]. Three studies performed miRNA transfection [25,31,33]. Dual-luciferase reporter assays and LPS stimulation were each utilized in two studies [25,31]. 

Types of Studies

The included studies represent a range of designs, including two cross-sectional case-control studies [32,34], a prospective observational study [35], and three in-vitro experimental studies [25,31,33].

Risk of Bias Assessment

The risk of bias in the studies included in this review was assessed using two established instruments and visualized using the Risk of Bias Visualization tool (ROBVIS) [36]. 

**Figure 2:**
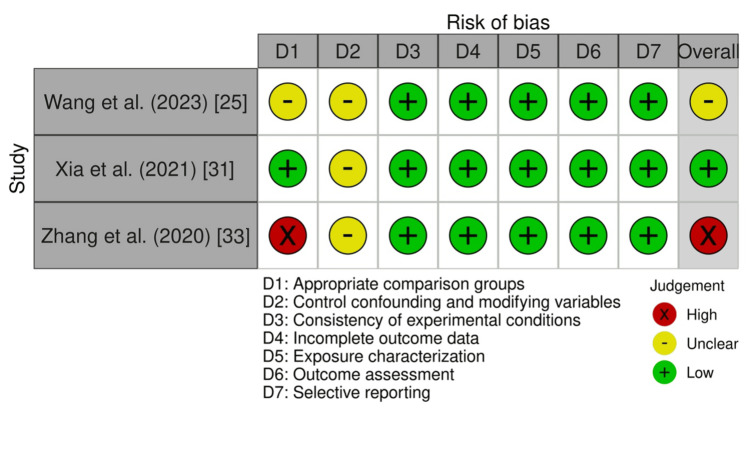
The Office of Health Assessment and Translation (OHAT) assessment results of the three included studies with bias evaluated for each domain and overall.

**Figure 3:**
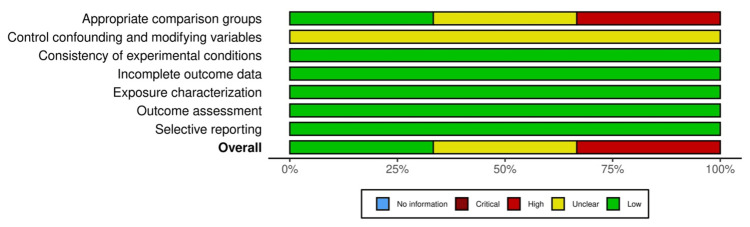
Summarized bias assessment results of the three included studies using the Office of Health Assessment and Translation (OHAT) risk of bias tool.

**Figure 4:**
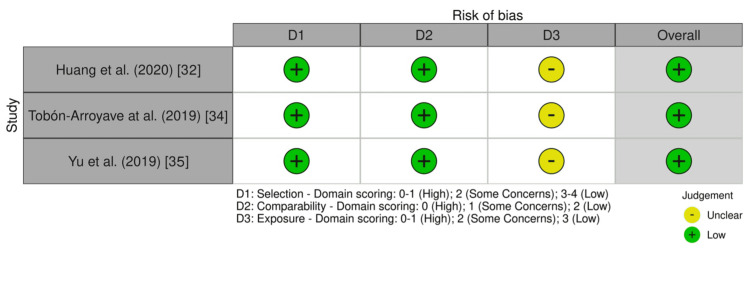
Newcastle–Ottawa Scale (NOS) assessment results of the three included studies with bias evaluated for each domain and overall.

**Figure 5:**
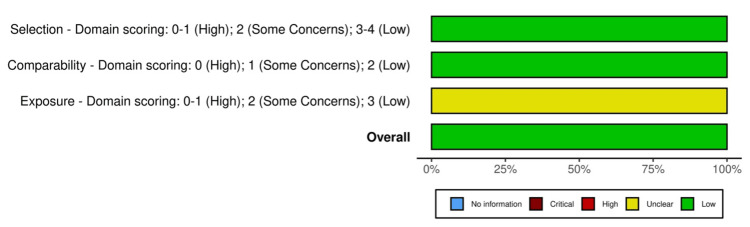
Summarized bias assessment results of the three included studies using the the Newcastle–Ottawa Scale (NOS) risk of bias tool.

Specifically, three in vitro experimental studies [25, 31, 33] were evaluated using an adapted version of the Office of Health Assessment and Translation (OHAT) risk of bias tool [37], in which only questions relevant to this study were selected (Figures 2-3). One study posed a low risk of bias [31], whereas one had unclear bias due to appropriate comparison groups (D1) and modifying variables (D2) [25]. One study posed a high risk of bias due to both appropriate control groups (D1) and control cofounding and modifying variables (D2) [33]. Additionally, two cross-sectional case-control studies [32, 34] and one prospective observational study [35] were assessed using the Newcastle-Ottawa Scale (NOS) [38] (Figures 4-5). As a result, three of the studies posed a low risk of bias [32, 34, 35].

Statistical Analysis

A systematic review was conducted, as meta-analysis was not achievable due to heterogeneity and variability in study design and outcomes, particularly in exosome sources, cargo, specific molecular targets, and the biological processes investigated. 

Results

Exosomal miRNA-Mediated Modulation of Pyroptosis and Macrophage Polarization in Periodontitis

Salivary exosomal miR-223-3p is significantly downregulated in periodontitis and plays a key role in suppressing pyroptosis, a programmed cell death process, in macrophages by targeting the NLRP3-caspase-1-GSDMD signaling axis [31]. The NLRP3 inflammasome is a key innate immune complex that drives IL-1 family cytokine production in periodontitis [31]. By targeting NLRP3, miR-223-3p suppresses activation of caspase-1 and reduces the release of the pro-inflammatory cytokines IL-1β and IL-6, as well as limiting GSDMD-mediated pyroptosis. In contrast, transfection with a miR-223-3p inhibitor led to significant upregulation of NLRP3, IL-6, and IL-1β mRNA in macrophages [31]. Therefore, it is suggested that reduced levels of salivary exosomal miR-223-3p may worsen periodontal inflammation by inflammasome activation and pyroptosis in macrophages [31]. Beyond its function, miR-223-3p also shows promise as a non-invasive salivary biomarker for assessing disease severity in periodontitis [31].

Furthermore, exosomes derived from PDLSCs exposed to an inflammatory environment promote pro-inflammatory macrophage polarization through miR-143-3p signaling [25]. Macrophages play a vital role in innate immunity and are capable of polarizing into M1 cells that promote inflammation or M2 cells that suppress it [25]. In periodontitis, the balance shifts toward M1 polarization [25]. Exosomes from inflamed PDLSCs carried elevated levels of miR-143-3p, which downregulated the expression of PI3Kγ in macrophages [25]. PI3Kγ is a critical checkpoint of immune regulation, and its inhibition by miR-143-3p led to downregulation of the PI3K/AKT pathway and activation of NF-κB signaling [25]. Consequently, M1 increased mRNA expression of IL-12 and MINCLE and protein expression of CCR7 and IL-1β, and M2 macrophage polarization declined, while reducing mRNA expression of CD163, IL-10, and protein expression of CD163 and MRC1 [25]. Blocking exosome release from inflammatory PDLSCs reversed this effect, downregulating M1 markers and enhancing M2-related expression, confirming that these exosomes drive M1 macrophage polarization [25]. The authors proposed that the elevated salivary exosomal PD-L1 mRNA observed in severe periodontitis could reflect an adaptive host mechanism to inhibit further tissue destruction. Measuring exosome-derived PD-L1 mRNA in saliva may help differentiate disease presence and severity, supporting its potential as a biomarker for periodontitis.

Exosome-Mediated Angiogenesis in Periodontal Inflammation

PDLSCs derived from patients with periodontitis secrete more exosomes enriched with increased amounts of vascular endothelial growth factor A (VEGFA), a key pro-angiogenic protein [33]. When applied to human umbilical vein endothelial cells (HUVECs), in vitro, these exosomes enhanced tube formation, indicating a role in inflammation-associated angiogenesis [33]. The exosomal transfer of VEGFA is regulated by miR-17-5p, a microRNA downregulated in inflamed PDLSCs [33]. When miR-17-5p was overexpressed in PDLSCs, it reduced the levels of VEGFA protein in their exosomes and suppressed endothelial tube formation [33]. Importantly, blocking exosome release from PDLSCs using GW4869 led to decreased tube formation in HUVECs and downregulated expression of angiogenic markers such as CD31 and VEGFA [33]. These findings may suggest that exosomes from inflamed PDLSCs contribute to vascular changes in periodontitis by transferring VEGFA, potentially playing a role in aberrant angiogenesis [33].

Exosomal Cargo and Immune Modulation in Periodontitis

Proteomic profiling of salivary exosomes from young adults with severe periodontitis revealed 26 proteins uniquely expressed in the disease group. GO and KEGG pathway analyses revealed that these proteins were predominantly associated with innate immune responses and complement activation [32]. Among them, complement components C6, C8A, and C8B, as well as chemokine ligand 28 (CCL28), were highlighted in the periodontitis group [32]. Western blot analysis confirmed that C6 was exclusively expressed in salivary exosomes from these individuals [32]. C6 is a member of the membrane attack complex (MAC), which forms during the terminal phase of complement activation [32]. Upon activation, the complement system mediates microbial phagocytosis, triggers the recruitment and activation of inflammatory cells, and induces direct lysis of microorganisms [32]. These findings suggest that salivary exosomes may not only reflect immune status but also contribute to immune activation through complement-related pathways [32]. Beyond the presence of immune-related proteins, salivary exosomes in periodontitis exhibit significantly reduced levels of the tetraspanins CD9 and CD81 [34]. These changes may impair exosomal function and suggest a broader role in regulating periodontal inflammation [34]. Patients with stages II, III, and IV exhibited lower CD9 concentrations than those with stage I periodontitis, while CD81 levels were significantly reduced in stages III and IV [34]. Similarly, by grade, individuals with grade C periodontitis had the lowest concentrations of both markers, indicating a correlation with disease progression [34]. However, only CD81 remained independently associated with periodontitis after adjusting for confounding variables, whereas the association of CD9 was attenuated by demographic covariables, limiting its diagnostic relevance [34].

Tetraspanins function as molecular facilitators that organize membrane proteins into specialized domains, thereby influencing cellular functions such as cell adhesion, migration, invasion, membrane fusion, signaling, and protein trafficking [34]. It is suggested that the decreased levels of CD9 and CD81 may disrupt molecular organization within the periodontal microenvironment, potentially contributing to tissue destruction and bone resorption in periodontitis [34]. Additionally, programmed death-ligand 1 (PD-L1) mRNA was significantly elevated in salivary exosomes and gingival tissues in patients with advanced periodontitis, with levels approximately tenfold higher than those observed in healthy controls [35]. Salivary exosomal PD-L1 levels also showed a strong correlation with gingival PD-L1 expression [35]. Notably, elevated exosomal PD-L1 expression was associated with an advanced stage of periodontitis, but not with other clinical variables, suggesting it could serve as an indicator of disease severity [35]. The authors hypothesized that the upregulation of PD-L1 in advanced periodontitis may represent a local mechanism to inhibit the destruction of inflammatory tissues [35]. The PICO analysis is presented in Table 3, and the summary of exosomal roles across major biological processes (pyroptosis, macrophage polarization, angiogenesis, and immune modulation) in periodontitis is presented in Table 4. The study size, primary outcomes, and statistical significance of the included studies are summarized in Table 5.

**Table 3:** PICO analysis table of the included studies.

Article	Year	Population	Intervention	Comparison	Outcome
Xia et al. [31]	2021	N=7; two groups: gingival tissues from stage III/IV periodontitis patients (n=4) and healthy individuals (n=3). Sample size not reported; two groups: saliva samples from periodontitis patients and healthy individuals.	Exosome isolation using an exosome isolation kit and centrifugation, exosome characterization by transmission electron microscopy and nanoparticle tracking analysis, THP-1 cell culture and P. gingivalis LPS stimulation, transfection of miRNA, qRT-PCR, Western blotting, dual-luciferase reporter assay, GEO database analysis, and immunohistochemical staining of gingival tissues.	Saliva and gingival tissues from healthy individuals	Salivary exosomal miR-223-3p was significantly downregulated in periodontitis. Inhibition of miR-223-3p increased inflammatory and pyroptosis markers (NLRP3, caspase-1, GSDMD, IL-1β, IL-6) in macrophages. NLRP3 and GSDMD-N were upregulated in periodontitis gingival tissue.
Wang et al. [25]	2023	N=10; two groups: primary human PDLSCs from healthy individuals (n=5) and patients with periodontitis (n=5)	LPS stimulation of PDLSCs, THP-1 cell culture and differentiation into M0 macrophages, exosome isolation using ultracentrifugation, exosome characterization by transmission electron microscopy, and Western blotting. Transwell coculture, miRNA microarray, qRT-PCR, GW4869 treatment, exosome uptake assay, inhibition assay, transfection assay, dual-luciferase assay, KEGG pathway enrichment analysis, 740Y-P treatment of miR-143-3p-transfected macrophages.	PDLSCs from healthy individuals	Exosomes from inflammatory PDLSCs increased M1 macrophage polarization and suppressed M2 markers, as shown by elevated IL-1β, IL-12, CCR7, and MINCLE expression, and reduced IL-10, CD163, and MRC1. miR-143-3p was identified as an exosomal cargo regulating this effect via PI3Kγ targeting, PI3K/AKT suppression, and NF-κB activation. Inhibition of exosome secretion reduced M1 polarization markers.
Zhang et al. [33]	2020	Sample size not reported; two groups: PDLSCs from patients with periodontitis and healthy individuals	Exosome isolation by ultracentrifugation, exosome characterization by transmission electron microscopy and scanning electron microscopy, flow cytometry, Western blotting, tube formation assay, Transwell coculture with HUVECs, miRNA transfection, dual-luciferase reporter assay, GW4869 inhibition of exosome secretion, and quantitative real-time PCR.	PDLSCs from healthy individuals	Exosomal VEGFA levels were increased due to reduced miR-17-5p expression in inflamed PDLSCs. Reduction of angiogenic effects (tube formation, CD31, VEGFA) upon blocking exosome secretion using GW4869 or overexpressing miR-17-5p.
Huang et al. [32]	2020	N=22; two groups: saliva from severe periodontitis patients (n=11) and healthy individuals (n=11)	Exosome isolation using an exosome isolation kit and centrifugation, transmission electron microscopy, BCA assay, Western blotting, LC-MS/MS protein identification, database search (UniProt), Gene Ontology enrichment analysis, protein-protein interaction network analysis (STRING).	Saliva from healthy individuals	Twenty-six exosomal proteins were uniquely identified in the periodontitis group. GO and KEGG analyses revealed enrichment in innate immune response, cytolysis, and complement activation pathways, notably involving C6, C8A, C8B, and CCL28. Western blotting confirmed expression of complement component C6 in salivary exosomes from the periodontitis group.
Tobón-Arroyave et al. [34]	2019	N=149; two groups: saliva samples from periodontitis patients (n=104) and healthy individuals (n=45)	Exosome isolation using an exosome isolation kit and centrifugation, transmission electron microscopy, and quantitative analysis of CD9 and CD81 tetraspanins by ELISA.	Saliva from healthy individuals	Salivary concentrations of exosome-associated tetraspanins CD9 and CD81 were significantly reduced in the periodontitis group compared to healthy controls. CD81 levels exhibited an independent association with periodontal disease. The lowest concentrations were observed in subjects with stage III-IV and grade C periodontitis.
Yu et al. [35]	2019	N=91; two groups: saliva from patients with chronic periodontitis (n=61) and healthy individuals (n=30), and gingival tissue samples were also collected from periodontitis patients (n=61)	Exosome isolation using an exosome isolation kit and centrifugation, exosome characterization by TEM, NTA, and immunoblotting, RT-qPCR for PD-L1 mRNA, THP-1 cell culture, Gene Set Enrichment Analysis (GSEA).	Saliva from healthy individuals	Salivary exosomal PD-L1 mRNA expression was significantly higher in the periodontitis group compared to controls and showed correspondence with gingival PD-L1 levels. Elevated exosomal PD-L1 was specifically associated with advanced disease stage, and not with other clinical parameters.

**Table 4:** Summary of exosomal roles across major biological processes (pyroptosis, macrophage polarization, angiogenesis, and immune modulation) in periodontitis. PDLSC: periodontal ligament stem cells

Biological Process	Exosomal Role
Pyroptosis	Exosomes act as key mediators of pyroptosis in periodontitis. Salivary exosomes with reduced miR-223-3p failed to inhibit the NLRP3–caspase-1–GSDMD pathway, leading to heightened macrophage pyroptosis and release of IL-1β and IL-6 in macrophages. Indicating that salivary exosomes actively modulate periodontal inflammation by inflammasome activation [31].
Macrophage polarization	Exosomes from inflamed PDLSCs promote pro-inflammatory M1 macrophage polarization via transferring miR-143-3p, which regulates the PI3K/AKT/NF-κB pathway. This increases pro-inflammatory cytokines (IL-12, IL-1β) and markers (CCR7, MINCLE) while reducing anti-inflammatory M2 markers (CD163, IL-10, MRC1), contributing to inflammatory progression in periodontitis [25].
Angiogenesis	Exosomes from inflamed PDLSCs promote angiogenesis by delivering VEGFA to endothelial cells, enhancing tube formation and angiogenic markers (CD31, VEGFA). This effect is amplified by reduced exosomal miR-17-5p in inflamed PDLSCs and blocked when exosome release is inhibited, highlighting their key role in inflammation-driven angiogenesis [33].
Immune modulation	Exosomes in periodontitis act as immune modulators by transporting key molecules that influence inflammatory activity [35]. Salivary exosomes enriched with complement proteins (C6, C8A, C8B) and CCL28 enhance innate immune and complement responses, while decreased exosomal tetraspanins (CD9, CD81) may weaken intercellular communication and contribute to tissue breakdown [32,34]. Elevated exosomal PD-L1 mRNA in advanced disease suggests an adaptive role in suppressing excessive inflammation, highlighting exosomes as active regulators of immune balance in periodontitis [35].

**Table 5:** Study size, primary outcomes and statistical significance of included studies. PDLSC: periodontal ligament stem cells; HUVEC: human umbilical vein endothelial cells; VEGFA: vascular endothelial growth factor A

Article	Year	Study size	Primary outcomes	Statistical significance
Xia et al. [31]	2021	N=7; two groups: gingival tissues from stage III/IV periodontitis patients (n=4) and healthy individuals (n=3). Sample size not reported; two groups: saliva samples from periodontitis patients and healthy individuals.	miR-223-3p ↓; NLRP3, caspase-1, GSDMD, IL-1β, IL-6 ↑	p<0.05 was considered statistically significant. miR-223-3p decreased (p < 0.1), NLRP3, caspase-1, GSDMD, IL-1β, and IL-6 increased (p < 0.05).
Wang et al. [25]	2023	N=10; two groups: primary human PDLSCs from healthy individuals (n=5) and patients with periodontitis (n=5)	miR-143-3p ↑; PI3Kγ ↓; PI3K/AKT signaling ↓; NF-κB signaling ↑; IL-1β ↑; IL-12 ↑; CCR7 ↑; MINCLE ↑; IL-10 ↓; CD163 ↓; MRC1 ↓; M1 polarization ↑; M2 polarization ↓.	p < 0.05 was considered statistically significant. Compared with healthy PDLSCs, periodontitis- and LPS-stimulated PDLSCs had reduced CD163, MRC1, and IL-10 expression (p < 0.05–0.01). CCR7 and IL-1β protein levels were elevated (p < 0.05-0.001). IL-12 increased in the periodontitis group (p < 0.01), MINCLE increased significantly with LPS stimulation (p < 0.001), and IL-8 changes were not significant (p > 0.05). CD163, MRC1, and IL-10 were significantly reduced in macrophages treated with exosomes from periodontitis or LPS-stimulated PDLSCs compared with healthy PDLSC exosomes (p < 0.05–0.01). IL-8 increased in both periodontitis and LPS-stimulated groups (p <0.05–0.01), while MINCLE and IL-12 rose significantly only with LPS-stimulated PDLSC exosomes (p < 0.05–0.001). miR-143-3p mimics decreased CD163, MRC1, and IL-10 expression (p < 0.05–0.001) and increased MINCLE and IL-12 (p < 0.05–0.01). Inhibition of exosome secretion in periodontitis PDLSCs increased CD163 (p < 0.05) and MRC1 (p < 0.01), and decreased IL-8 (p < 0.01). IL-10, MINCLE, and IL-12 showed no significant difference (p > 0.05). Inhibition of LPS-stimulated PDLSCs increased CD163 (p < 0.001) and MRC1 (p < 0.05), and decreased MINCLE and IL-12 (p < 0.05); IL-8 and IL-10 were not significant (p > 0.05).
Zhang et al. [33]	2020	Sample size not reported; two groups: PDLSCs from patients with periodontitis and healthy individuals	TSG101 ↑; CD63 ↑; VEGFA ↑; miR-17-5p ↓; HUVEC ↑.	p < 0.05 was considered statistically significant. TSG101 and CD63 expression increased in inflamed PDLSCs (p < 0.01). Tube formation in HUVECs treated with inflamed PDLSCs or their exosomes increased (p < 0.01). miR-17-5p expression decreased under inflammatory conditions, and its inhibition increased VEGFA protein levels (p < 0.01). The VEGFA expression was higher in exosomes derived from inflamed PDLSCs than control group (p < 0.01).
Huang et al. [32]	2020	N=22; two groups: saliva from severe periodontitis patients (n=11) and healthy individuals (n=11)	C6, C8A, C8B, CCL28, SAA1.	p<0.05 was considered statistically significant. GO/KEGG enrichment was highly significant (p < 0.001 to p < 1×10⁻¹⁰).
Tobón-Arroyave et al. [34]	2019	N=149; two groups: saliva samples from periodontitis patients (n=104) and healthy individuals (n=45)	CD9↓; CD81↓.	p<0.05 was considered statistically significant. CD9 decreased (p = 0.010), not significant after adjustment (p > 0.05), CD81 decreased (p < 0.001), remained significant after adjustment (p < 0.05). CD81 exosome levels remained independently associated with disease status (p < 0.05). An interaction between CD9 exosome concentration and age was observed ( p < 0.001). CD9 and CD81 salivary exosomes showed weak but significant negative correlations with PD, CAL, and extent (p = 0.001–0.029)
Yu et al. [35]	2019	N=91; two groups: saliva from patients with chronic periodontitis (n=61) and healthy individuals (n=30), gingival tissue samples were also collected from periodontitis patients (n=61)	PD-L1 ↑	P<0.05 was considered statistically significant. Mean salivary exosomal and gingival PD-L1 mRNA expression in periodontitis patients were both approximately 10-fold higher than in control subjects (p < 0.001). Salivary and gingival PD-L1 mRNA levels were positively correlated (p < 0.001). High PD-L1 expression was associated with advanced periodontitis stage (p = 0.005).

Discussion

This review synthesizes current scientific evidence to highlight the multifaceted role of exosomes as central mediators of host-pathogen interactions in the pathogenesis of periodontitis. The analyzed studies demonstrate that exosomes contribute to disease progression through diverse mechanisms, including immune cell modulation, inflammatory signaling, and tissue remodeling [25,31-35]. These effects involve macrophage polarization, pyroptosis, angiogenesis, and the transport of disease-related proteins and regulatory molecules [25,31-35]. Recent research has expanded the understanding of exosome-mediated mechanisms beyond periodontitis, with numerous studies investigating their roles in other inflammatory and systemic diseases.

Beyond periodontal disease, exosomes have been studied in malignancies, particularly oral squamous cell carcinoma (OSCC) and other head and neck squamous cell carcinomas (HNSCC), where they play important roles in immune modulation and intercellular communication that drive disease progression [39-41]. 

One of the findings in this review was the upregulation of programmed death-ligand 1 (PD-L1) mRNA in salivary exosomes and gingival tissues in patients with advanced periodontitis, which possibly reflected an adaptive mechanism to modulate inflammation and prevent excessive tissue destruction, as well as implicating disease severity [35]. A similar pattern of increased exosomal PD-L1 has been reported in OSCC and other HNSCCs. Plasma-derived exosomes carrying high levels of PD-L1, which are called PD-L1+, were significantly elevated in HNSCC patients with active disease, advanced tumor stage, and lymph node metastasis. These exosomes suppressed CD8+ T cell activation, shown by reduced CD69 expression, an effect reversed by PD-1 blockade, highlighting their immunosuppressive function [42]. 

In support of the findings, HNSCC tumor-derived exosomes carrying PD-L1 can induce the differentiation of regulatory T cells (aTregs) and M2 macrophages and further promote immunosuppression through a positive feedback loop between these cell types [43,44]. PD-L1-positive exosomes in mouse models enhanced tumor growth and suppressed antitumor immunity, while knockout of PD-L1 on exosomes led to reduced tumor growth and amplified immune responses [43]. The significance of exosomal PD-L1 is further illustrated in OSCC, where endoplasmic reticulum (ER) stress, which is a disturbance in the ER environment caused by factors such as nutrient deprivation, oxidative stress, or DNA damage, activates the unfolded protein response (UPR) [45,46]. In tumor cells, this response helps restore cellular homeostasis and shapes a microenvironment that supports tumor survival and progression [45]. It has also been shown to drive the release of PD-L1-enriched exosomes from tumor cells. These exosomes are taken up by macrophages, inducing polarization toward the immunosuppressive M2 type, both in vitro and in vivo [47]. This mechanism highlights how ER stress in tumor cells promotes an immunologically evasive microenvironment via exosome-mediated signaling. 

Conversely, in this review, exosome-mediated macrophage polarization in periodontitis was characterized by a shift toward the pro-inflammatory M1 phenotype. This was driven by miR-143-3p from inflamed PDLSCs, which activated the PI3K/AKT/NF-κB pathway and promoted alveolar bone loss and periodontal ligament inflammation in vivo [25]. In contrast, as previously mentioned, the exosome-driven response in cancer primarily promotes an anti-inflammatory, immunosuppressive shift toward M2 macrophage polarization. Similar findings have also been observed in exosome studies on tongue squamous cell carcinoma [48] and laryngeal squamous cell carcinoma [49], further supporting the tumor-promoting role of M2 polarization across various head and neck cancer subtypes. However, not all malignancies display a uniform shift towards M2 polarization. For instance, exosomes derived from Human Papillomavirus (HPV)-positive HNSCC induce M1 polarization of macrophages, rather than M2 [50]. Specifically, exosomal miR-9, which is enriched in HPV+ HNSCC, was shown to promote M1 macrophage polarization by downregulating PPARδ, thereby increasing tumor radiosensitivity and improving prognosis [51]. The distinct pattern of macrophage polarization observed in HPV+ HNSCC may be due to the underlying inflammatory nature of HPV infection. Similar to periodontitis, it involves chronic inflammation and altered immune regulation [52]. Studies have reported a correlation between HPV infection and periodontitis, suggesting that persistent periodontal inflammation may increase susceptibility to oral HPV infection, and vice versa [53]. This overlapping inflammatory microenvironment may partly explain the shared pro-inflammatory exosomal signaling observed in both conditions. 

In this review, the regulatory role of exosomal miR-223-3p in periodontitis was also highlighted, specifically its ability to suppress NLRP3 inflammasome-mediated pyroptosis and the release of pro-inflammatory cytokines such as IL-1β and IL-6 in macrophages [31]. Notably, miR-223-3p is significantly downregulated in both periodontitis and OSCC. Functionally, miR-223-3p suppresses the proliferation and migration of OSCC cells and promotes apoptosis [54]. In both diseases, inhibition or downregulation of miR-223-3p leads to pathogenic processes, such as excessive inflammation and pyroptotic cell death in periodontitis, and malignant cell proliferation and tumor survival in OSCC [31,54]. Thus, miR-223-3p can exert both anti-inflammatory effects in chronic inflammation and anti-tumor effects in cancer, depending on the tissue environment and disease context.

Angiogenesis normally supports host defense by enhancing immune cell access and promoting tissue repair. However, in chronic periodontitis, this process becomes dysregulated, facilitating excessive inflammatory cell infiltration and vascular dysfunction [55]. As a result, pathological angiogenesis contributes to periodontal pocket formation, edema, and tissue damage [55]. Exosomes have been implicated in this shift from protective to pathological angiogenesis. Research by Zhang et al. (2020) has demonstrated that exosomes from inflamed PDLSCs carry elevated VEGFA levels, modulated by miR-17-5p, and promote endothelial tube formation [33]. While in periodontitis, pathological angiogenesis contributes to tissue breakdown and sustained inflammation, in cancer, this process is often amplified to support tumor survival and expansion. In OSCC, tumor-derived exosomes enhance angiogenesis by delivering pro-angiogenic molecules, such as miR-130b-3p, miR-221, miR-421, and miR-205-5p [56-59]. These vesicles stimulate HUVEC proliferation, migration, and tube formation, and in some cases, promote tumor growth and neovascularization in vivo [56-59]. In a research by He et al., Huang CY et al., Huang W et al., and Yan et al., the pro-angiogenic effects of these exosomal miRNAs were mediated by targeting and regulating angiogenesis-associated genes and proteins, including PIK3R1 (miR-221), HS2ST1 (miR-421), PTEN (miR-130b-3p), and AMOT (miR-205-5p), thereby modulating endothelial cell behavior and the tumor microenvironment [56-59]. While exosome-mediated angiogenesis contributes to tissue destruction and sustained inflammation in periodontitis, in OSCC, the same process facilitates tumor growth and neovascularization, highlighting how similar mechanisms can have different effects depending on the disease context.

Additionally, this review found that salivary exosomal tetraspanins CD81 and CD9 were reduced in advanced periodontitis (stages II-IV, grade C), although only CD81 remained independently associated. Given their role in organizing membrane proteins into functional microdomains [60], lower CD81/CD9 may compromise membrane signaling and contribute to tissue destruction and bone loss [34]. In OSCC tissue, CD9 is also downregulated (42% of OSCC cases) is linked to lymph-node metastasis and shorter disease-free and overall survival, whereas CD81 is not detectable on normal or malignant keratinocyte membranes, suggesting limited involvement in the mechanisms of OSCC [61]. Similar associations between reduced CD9, greater invasiveness, and metastatic potential are reported in laryngeal squamous cell carcinoma (LSCC) and esophageal squamous cell carcinoma (ESCC) [62]. Beyond the head and neck, in hepatocellular carcinoma, tetraspanins CD9 and CD81 are tumor suppressive: CD81 is frequently lost in tumor tissue, and reduced CD9/CD81 expression correlates with advanced stage and poorer outcomes [63].

Beyond cancer, diabetes offers an important context for exosome effects in periodontitis. Diabetes, characterized by chronic hyperglycemia, impairs host defense and tissue repair and increases susceptibility to periodontal and other oral diseases [64]. Because key inflammatory pathways are shared and bidirectional, diabetes and periodontitis often co-occur and can worsen each other [65]. Epidemiologically, individuals with periodontitis show higher diabetes prevalence and odds than periodontally healthy controls [66]. As outlined in this review, exosomal cargo drives periodontitis and, in diabetes, appears further skewed, amplifying inflammation. In type 2 diabetes, salivary exosomal miR-25-3p is elevated and increases the population of IL-17-producing cells, which leads to periodontal inflammation and bone loss [67]. In addition, salivary exosomal miR-25-3p was higher in elderly patients with type 2 diabetes mellitus (T2DM), osteoporosis, and periodontitis than in those with T2DM and osteoporosis only, indicating periodontitis further elevates miR-25-3p in this group [68]. Furthermore, in a diabetic model, bone marrow-derived macrophage exosomes show elevated miR-381-3p, which in gingival epithelial cells suppresses NR5A2, reduces ATG7-dependent autophagy, and heightens NLRP3/IL-1β signaling, thereby worsening periodontal inflammation [69]. 

Limitations and Future Directions

Nevertheless, while the emerging data are suggestive, further studies are needed to clarify exosomes' role in host-pathogen interactions in periodontitis. Because of the heterogeneity of study designs and outcomes, findings were synthesized qualitatively rather than quantitatively. The evidence base remains constrained by a small number of studies, limited sample sizes, and heavy reliance on animal and in vitro models, with limited direct evidence from human periodontitis patients. Future studies should include larger sample sizes and place greater emphasis on exosomes derived from human tissues rather than animal or cell-line models. Moreover, more studies should focus not only on therapeutic applications but also on the underlying host-pathogen and cell-to-cell interactions mediated by exosomes, as highlighted by the difficulties encountered in identifying suitable studies for this review.

## Conclusions

Exosome biogenesis and their ability to transport diverse molecular cargo and influence recipient cell behavior could emphasize their role as key mediators of intercellular communication in periodontal inflammation. Salivary and PDLSC-derived exosomal miR-223-3p and miR-143-3p modulate pyroptosis and macrophage polarization, thereby promoting pro-inflammatory responses in periodontitis. In addition, exosomal VEGFA from inflamed PDLSCs contributes to aberrant angiogenesis in periodontal tissues. Salivary exosomal cargo in periodontitis includes complement components C6, C8A, C8B, and chemokine CCL28, together with decreased CD9/CD81 and elevated PD-L1 mRNA, which may indicate altered immune regulation and correlate with disease severity.
